# The green impact: bacterioplankton response toward a phytoplankton spring bloom in the southern North Sea assessed by comparative metagenomic and metatranscriptomic approaches

**DOI:** 10.3389/fmicb.2015.00805

**Published:** 2015-08-11

**Authors:** Bernd Wemheuer, Franziska Wemheuer, Jacqueline Hollensteiner, Frauke-Dorothee Meyer, Sonja Voget, Rolf Daniel

**Affiliations:** ^1^Genomic and Applied Microbiology and Göttingen Genomics Laboratory, Institute of Microbiology and Genetics, Georg-August-University GöttingenGöttingen, Germany; ^2^Department for Crop Sciences, Georg-August-University GöttingenGöttingen, Germany

**Keywords:** bacterioplankton, metagenomics, metatranscriptomics, algal bloom, functional changes, *Planktomarina temperata*, SAR92

## Abstract

Phytoplankton blooms exhibit a severe impact on bacterioplankton communities as they change nutrient availabilities and other environmental factors. In the current study, the response of a bacterioplankton community to a *Phaeocystis globosa* spring bloom was investigated in the southern North Sea. For this purpose, water samples were taken inside and reference samples outside of an algal spring bloom. Structural changes of the bacterioplankton community were assessed by amplicon-based analysis of 16S rRNA genes and transcripts generated from environmental DNA and RNA, respectively. Several marine groups responded to bloom presence. The abundance of the *Roseobacter* RCA cluster and the SAR92 clade significantly increased in bloom presence in the total and active fraction of the bacterial community. Functional changes were investigated by direct sequencing of environmental DNA and mRNA. The corresponding datasets comprised more than 500 million sequences across all samples. Metatranscriptomic data sets were mapped on representative genomes of abundant marine groups present in the samples and on assembled metagenomic and metatranscriptomic datasets. Differences in gene expression profiles between non-bloom and bloom samples were recorded. The genome-wide gene expression level of *Planktomarina temperata*, an abundant member of the *Roseobacter* RCA cluster, was higher inside the bloom. Genes that were differently expressed included transposases, which showed increased expression levels inside the bloom. This might contribute to the adaptation of this organism toward environmental stresses through genome reorganization. In addition, several genes affiliated to the SAR92 clade were significantly upregulated inside the bloom including genes encoding for proteins involved in isoleucine and leucine incorporation. Obtained results provide novel insights into compositional and functional variations of marine bacterioplankton communities as response to a phytoplankton bloom.

## Introduction

Bacteria are major drivers in cycling of nitrogen, carbon, and other elements in marine ecosystems (Azam et al., 1983; Arrigo, 2005; DeLong and Karl, 2005). More than 50% of organic matter produced by phytoplankton is remineralized by marine bacteria (Cole et al., 1988; Karner and Herndl, 1992; Ducklow et al., 1993). Therefore, bacteria play an important role during and after bloom events as large amounts of organic matter are generated by primary production (Azam, 1998).

Recent studies investigating bacterioplankton communities during phytoplankton blooms revealed that community structures and diversity were highly affected (Teeling et al., 2012; Liu et al., 2013; Wemheuer et al., 2014). Observed patterns were correlated to changes of nutrient concentrations and other environmental factors such as water depth or algal species (Fandino et al., 2001; Pinhassi et al., 2004; Grossart et al., 2005; Teeling et al., 2012; Liu et al., 2013; Wemheuer et al., 2014; Gomes et al., 2015). Consequently, understanding the dynamics and interactions between bacterial communities and phytoplankton blooms is crucial to validate the ecological impact of bloom events.

One region with annually recurring spring phytoplankton blooms is the North Sea, a typical coastal shelf sea of the temperate zone. Shelf seas are highly productive due to the continuous nutrient supply by rivers. During the last 40 years, the North Sea and in particular its southern region, the German Bight, underwent high nutrient loading and warming (McQuatters-Gollop et al., 2007; Wiltshire et al., 2008, 2010). Recent studies aimed at understanding bacterial responses to phytoplankton blooms in the North Sea (Alderkamp et al., 2006; Teeling et al., 2012; Wemheuer et al., 2014). A dynamic succession of distinct bacterial clades before, during, and after bloom events in the North Sea was observed in several investigations (Alderkamp et al., 2006; Alonso and Pernthaler, 2006a,b; Teeling et al., 2012). The results indicate that specialized populations occupy ecological niches provided by phytoplankton-derived substrates (Teeling et al., 2012). Klindworth et al. (2014) investigated the diversity and activity of marine bacterioplankton during the same bloom event applying metatranscriptomic techniques. They showed that members of the *Rhodobacteraceae* and SAR92 clade exhibited high metabolic activity levels. However, recent research focused mainly on changes of community structure as response to phytoplankton blooms, but functional changes and their resulting ecological impacts have been rarely studied. In addition, larger comparative metagenomic and metatranscriptomic studies investigating structural and functional changes of the bacterioplankton during the bloom event are lacking.

In a previous study, we investigated structural differences of the active bacterioplankton community as response toward a *Phaeocystis globosa* bloom in the southern North Sea in spring 2010 mainly by 16S rRNA pyrotag sequencing (Wemheuer et al., 2014). This microalgae has a cosmopolitan distribution (Schoemann et al., 2005) and is considered to be responsible for harmful algal blooms (Veldhuis and Wassmann, 2005). These blooms have been observed in many marine environments, including the coast of the eastern English Channel, the southern North Sea and the south coast of China (Schoemann et al., 2005). We found that the phytoplankton spring bloom impacted bacterioplankton community structures and the abundance of certain bacterial groups significantly. For example, the *Roseobacter* RCA cluster and the SAR92 clade were significantly more abundant in the bloom at active community level. For the current study, more than 500 million sequences derived from direct sequencing of environmental DNA and rRNA depleted RNA were added to obtain functional insights into the bloom event. Metatranscriptomic data was mapped on the assembled metagenomic and metatranscriptomic data sets and on the genomes of abundant marine bacteria, e.g., *P. temperata* RCA23, a member of the *Roseobacter* RCA cluster. In addition, 16S rRNA genes and transcripts were studied by pyrotag sequencing to obtain insights into structural dynamics of the total and active bacterioplankton community, respectively. The comprehensive experimental design and method combination of this study sheds new light on ecological roles and functions of single members of the bacterioplankton community and the entire community.

## Materials and methods

### Sampling and sample preparation

**Figure 1:**
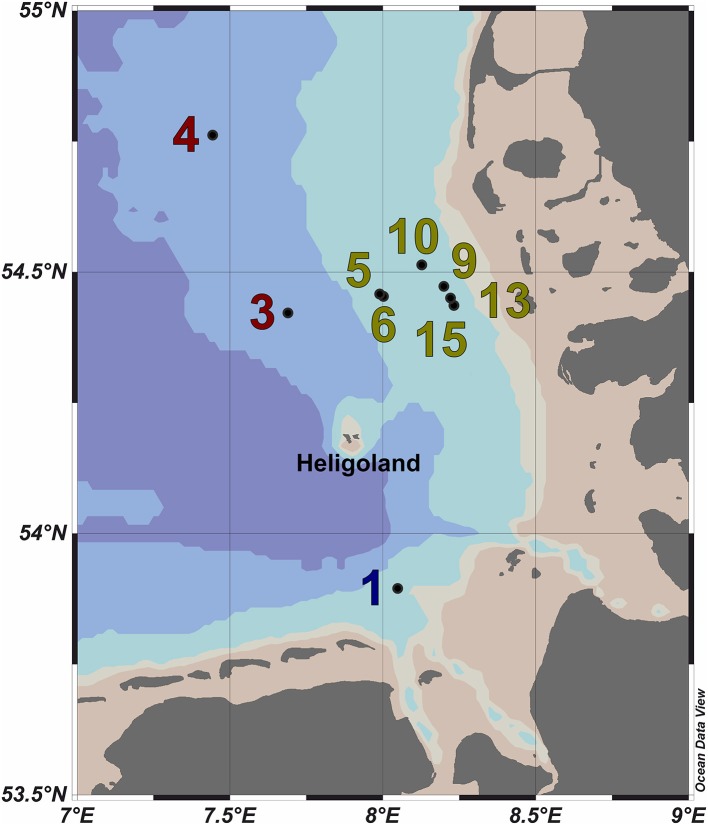
**Map of the German Bight showing the locations of the nine sampling stations visited in May 2010**. Stations inside the examined phytoplankton bloom are depicted in green; those outside the bloom in red. Station 1 is depicted in blue as it was located in a bloom outside of the area of the examined bloom. Shading of the water masses refers to bottom depth. The map was generated using the Ocean Data View software package [version 4.7.2; Schlitzer, 2015 (http://odv.awi.de/)].

Ten water samples for bacterioplankton analyses were collected in the southern North Sea at nine stations in and outside of a *P. globosa* bloom in May 2010 (Figure 1; Table 1). Six samples were taken in the presence of a phytoplankton bloom (3a, 3b, and 4) and three in bloom absence (5–15). One sample was taken near to a river outfall (1). Stations inside the bloom were located by satellite images and are characterized by their increased chlorophyll content. Note that sample 9 was taken in the bloom area and is considered as a bloom sample despite its relative low chlorophyll content. Sampling and filtration were performed as described previously (Wemheuer et al., 2014). In brief, obtained water samples were initially filtered using a 10 μm nylon net filter and 2.7 μm glass fiber filter. Bacterioplankton was subsequently harvested from a prefiltered 1 l sample on a filter sandwich consisting of a glass fiber and 0.2 μm polycarbonate filter (47 mm diameter). Samples for community analysis were stored at −80°C until further analysis. Several environmental parameters such as chlorophyll *a* (Chl *a*), particulate organic nitrogen (PON), salinity, temperature, and nitrate content were determined as described previously (Wemheuer et al., 2014) (Table 2).

**Table 1:** **Sampling site characteristics**.

**Sample**	**Ship station**	**Origin**	**Date (mm/dd/yyyy)**	**Latitude (°N)**	**Longitude (°E)**	**Depth (m)**	**Bottom depth (m)**
1	655	River outfall	05/25/2010	53.8955	8.0496	2	15.5
3a	657a	No bloom	05/26/2010	54.4223	7.6833	2	22.1
3b	657b	No bloom	05/26/2010	54.4223	7.6833	12	22.1
4	658	No bloom	05/26/2010	54.7626	7.4463	2	20
5	659	Bloom	05/26/2010	54.4575	7.9893	9	12.5
6	660	Bloom	05/27/2010	54.4542	8.0018	2	12.5
9	664	Bloom	05/28/2010	54.4733	8.1972	2	12
10	665	Bloom	05/28/2010	54.5135	8.128	2	11
13	668	Bloom	05/29/2010	54.4365	8.2328	10	12
15	671	Bloom	05/30/2010	54.449	8.22	2	12

**Table 2:** **Environmental parameters measured for the 10 water samples**.

**Sample**	**Temperature**	**Salinity**	**Fluorescence**	**Transmission**	**Density**	**Chlorophyll a**	**Phaeopigment**	**Suspended particulate matter**	**Particulate organic carbon**	**Particulate organic nitrogen**	**Nitrate**	**Nitrite**	**Mono-nitrogen oxides**	**Phosphate**
	**(°C)**	**(psu)**	**(FU)**	**(%)**	**(g/l)**	**(μg/L)**	**(μg/L)**	**(μg/L)**	**(μg/L)**	**(μg/L)**	**(μM)**	**(μM)**	**(μM)**	**(μM)**
**RIVER OUTFALL**														
1	11.09	30.24	1.21	57.2	1023.1	4.38	2.11	9.8	997.4	152.1	8.46	0.19	8.65	0.15
**NON-BLOOM**														
3a	9.43	31.42	0.2	87.98	1024.27	1.12	0.25	4.6	291.2	43	7.395	0.25	7.65	0.02
3b	8.18	32.01	0.77	84.83	1024.93	3.37	1.08	7.15	496.4	82.2	5.9	0.27	6.17	0.04
4	9.73	32.71	0.49	81.23	1025.2	2.55	0.37	6.15	290.4	46.9	6.17	0.24	6.41	0.03
**BLOOM**														
5	10.8	30.64	2.76	60.14	1023.5	11.45	7.03	3.27	1673	213.6	5.03	0.24	5.27	0.1
6	10.83	30.65	1.89	72.78	1023.4	7.34	2.77	11.3	728.2	106.2	9.11	0.42	9.53	0.08
9	10.9	30.76	1.14	87.28	1023.5	2.19	0.6	3.2	367.7	49	4.94	0.24	5.18	0.02
10	11.4	31.11	2.27	74.79	1023.7	6.93	2.1	7.5	737.5	95.3	3.685	0.29	3.98	0.07
13	11.83	31.18	2.8	67.83	1023.7	5.53	3.16	9.91	936.5	123.5	2.085	0.21	2.29	0.1
15	11.7	31.04	NA(^*^)	76.59	1023.6	5.33	1.58	6.25	624.4	83.9	2.97	0.3	3.27	0.08

* *Not measured due to fluorometer malfunction*.

*Although not exhibiting high chlorophyll a values, sample 9 is considered as a bloom sample as it was taken in the bloom area*.

### Extraction and purification of environmental DNA and RNA

Environmental DNA and RNA were co-extracted from the filter sandwich as described by Weinbauer et al. (2002). DNA and RNA were subsequently purified employing the peqGOLD gel extraction kit (Peqlab, Erlangen, Germany) and the RNeasy Mini Kit (Qiagen, Hilden, Germany), respectively, as recommended by the manufacturers. Residual DNA was removed from RNA samples and its absence was confirmed according to Wemheuer et al. (2012).

To assess bacterioplankton community structures, DNA-free RNA was directly converted to cDNA employing the SuperScript® III reverse transcriptase (Invitrogen™, Carlsbad, USA) using a primer specific for the conserved region downstream to variable region 6 of the 16S rRNA (1063r 5′-CTCACGRCACGAGCTGACG-3′). The reaction mixture (20 μl) contained 4 μl of five-fold reaction buffer, 500 μM of each of the four desoxynucleoside triphosphates, 5 mM DTT, 1 μM of the reverse primer, 1 U RiboLock™ RNase Inhibitor (Thermo Fisher Scientific, Schwerte, Germany), 200 U of the reverse transcriptase and approximately 100 ng DNA-free RNA. The reaction was incubated at 55°C for 1 h and subsequently inactivated by incubation at 70°C for 15 min. To remove the RNA in the RNA/DNA hybrids, 2.5 U RNase H (Thermo Fischer Scientific) were added and the reaction incubated at 37° for 15 min followed by inactivation at 65°C for 10 min. Obtained cDNA was subsequently subjected to 16S rRNA gene PCR (as described below). To assess community functions, environmental mRNA was enriched from total RNA using the RiboMinus™ transcriptome isolation kit for Bacteria (Invitrogen™, Carlsbad, USA) with one modification. The initial denaturation of RNA was performed at 70°C for 10 min. RNA was subsequently converted to cDNA employing the SuperScript™ double-stranded cDNA synthesis kit (Invitrogen™) with slight modifications according to Wemheuer et al. (2014). The Göttingen Genomics Laboratory determined the sequences of the extracted DNA and enriched mRNA-derived cDNA using a Roche 454 GS-FLX+ pyrosequencer with titanium chemistry (Roche, Mannheim, Germany) and an Illumina Genome Analyzer IIx (San Diego, USA), respectively (Table 3).

**Table 3:** **Sequence statistics of the quality trimmed metagenome and metatranscriptome data used in this study**.

**Sample**	**Technology**	**Single run/Paired End/Unpaired (SR/PE/UP)**	**Type**	**Sequence number**	**Average read length**	**Total basepairs**
1^*^	454 FLX+	SR	gDNA	338,735	252.66	85,583,953
1^*^	454 FLX+	SR	mRNA	421,864	275.62	116,273,765
1	Illumina GIIA	PE	gDNA	21,186,566	104.44	2,212,675,749
1	Illumina GIIA	UP	gDNA	362,317	83.16	30,128,732
1	Illumina GIIA	SR	mRNA	20,782,683	73.56	1,528,826,535
3a	Illumina GIIA	PE	gDNA	12,578,554	104.29	1,311,791,324
3a	Illumina GIIA	SR	gDNA	10,843,677	104.93	1,137,876,968
3a	Illumina GIIA	UP	gDNA	33,298,482	103.62	3,450,551,533
3a	Illumina GIIA	SR	mRNA	24,527,136	72.90	1,787,928,626
3b	Illumina GIIA	PE	gDNA	19,210,244	105.28	2,022,514,254
3b	Illumina GIIA	SR	gDNA	8,438,275	104.14	87,878,7402
3b	Illumina GIIA	UP	gDNA	9,783,518	101.24	99,052,5636
3b	Illumina GIIA	SR	mRNA	27,810,866	73.14	2,034,036,519
4^*^	454 FLX+	SR	mRNA	186,132	255.15	47,492,480
4	Illumina GIIA	PE	gDNA	29,884,508	100.60	3,006,238,128
4	Illumina GIIA	UP	gDNA	642,734	75.36	48,433,894
4	Illumina GIIA	SR	mRNA	14,014,013	73.37	1,028,189,524
5^*^	454 FLX+	SR	gDNA	391,106	252.93	98,922,278
5^*^	454 FLX+	SR	mRNA	490,182	274.96	134,779,442
5	Illumina GIIA	PE	gDNA	25,109,444	104.71	2,629,205,182
5	Illumina GIIA	UP	gDNA	17,758,425	102.47	1,819,684,124
5	Illumina GIIA	PE	mRNA	35,492	69.05	2,450,563
5	Illumina GIIA	SR	mRNA	11,759,937	72.22	849,301,842
5	Illumina GIIA	UP	mRNA	8,714,379	103.34	900,565,197
6	Illumina GIIA	PE	gDNA	21,372,550	105.26	2,249,604,814
6	Illumina GIIA	UP	gDNA	32,730,493	103.10	3,374,578,797
6	Illumina GIIA	SR	mRNA	14,893,714	72.25	1,076,040,735
9	Illumina GIIA	PE	gDNA	24,735,216	103.57	2,561,948,071
9	Illumina GIIA	UP	gDNA	539,723	82.72	44,644,280
9	Illumina GIIA	PE	mRNA	43,792	70.96	3,107,595
9	Illumina GIIA	SR	mRNA	15,900,375	72.46	1,152,110,018
9	Illumina GIIA	UP	mRNA	9,779,257	104.90	1,025,839,134
10	Illumina GIIA	PE	gDNA	20,787,102	101.19	2,103,509,139
10	Illumina GIIA	UP	gDNA	662,370	81.29	53,843,938
10	Illumina GIIA	PE	mRNA	87,236	71.04	6,197,063
10	Illumina GIIA	SR	mRNA	9,005,445	72.68	654,542,993
10	Illumina GIIA	UP	mRNA	13,299,109	104.04	1,383,633,962
13^*^	454 FLX+	SR	mRNA	10,273	254.59	2,615,404
13	Illumina GIIA	PE	gDNA	28,055,686	102.21	2,867,561,091
13	Illumina GIIA	UP	gDNA	858,735	81.56	70,037,612
13	Illumina GIIA	SR	mRNA	26,455,179	72.54	1,919,045,434
15	Illumina GIIA	PE	gDNA	22,984,400	101.81	2,340,126,872
15	Illumina GIIA	UP	gDNA	521,111	81.11	42,269,455
15	Illumina GIIA	SR	mRNA	22,592,228	71.65	1,618,686,787
17^*^	454 FLX+	SR	mRNA	109,911	235.46	25,879,456
Total				563,993,174	93.49153275	52,728,586,300

*Only Illumina-derived data not generated in a paired-end run was used in the metatranscriptomic mapping approach. Seqeuncing was performed using a Roche 454™ GS-FLX+ pyrosequencer with titanium chemistry and an Illumina Genome Analyzer IIx, respectively*.

* *Published under accession number SRA061816*.

### Processing and analysis of metagenomic and metatranscriptomic datasets

Generated metagenomic and metatranscriptomic datasets were initially processed according to Voget et al. (2014). Briefly, fastq files derived from Illumina sequencing were processed employing the Trimmomatic tool version 0.30 (Bolger et al., 2014). Sff files derived from pyrosequencing were converted to fastq files prior to quality filtering. Afterwards, all sequences were combined and assembled at different kmer values (29–109 in 10 bp steps) with Velvet and Metavelvet (Zerbino and Birney, 2008; Namiki et al., 2012). Subsequently, all obtained contigs were joined and resulting sequences were dereplicated employing Usearch version 7.0.190 (Edgar, 2010). Open reading frames (ORFs) were predicted for all remaining contigs using Prodigal version 2.6 (Hyatt et al., 2010). Short contigs (< 150 bp) were removed prior to further analysis.

As the metagenomic and metatranscriptromic datasets are likely to contain algal-derived sequences, we subtracted bacterial genes by blast alignment (Camacho et al., 2009) against 15 reference genomes of abundant marine lineages (Table 4) obtained from the integrated microbial genomes (IMG) platform (Markowitz et al., 2012). Genomes of abundant phylogenetic groups as found in the 16S rRNA analysis were chosen for this additional filtering step. Only sequences with an *e*-value below 0.001 were used in the subsequent analysis. Remaining ORFs were further classified employing UProC version 1.2 in protein mode (Meinicke, 2015).

**Table 4:** **Genomes retrieved from the Integrated Microbial Genomes (IMG) database**.

**IMG name**	**IMG Taxon ID**	**Genome Size (bp)**	**Gene Count**	**Phylum/Proteobacterial class**	**Marine Group/Genus**
*Polaribacter* sp. Hel_I_88	2,558,860,973	3,996,527	3552	*Bacteroidetes*	*Polaribacter*
*Marivirga tractuosa* H-43, DSM 4126	649,633,065	4,516,490	3857	*Bacteroidetes*	*Marivirga*
*Gammaproteobacteria* bacterium MOLA455	2,590,828,686	2,605,026	2374	*Gammaproteobacteria*	SAR92 clade
*Planktomarina temperata* RCA23, DSM 22400 (RCA23)	2,548,877,138	32,88,122	3101	*Alphaproteobacteria*	*Roseobacter* RCA
*Betaproteobacteria* bacterium MOLA814	2,590,828,684	2,859,706	2733	*Betaproteobacteria*	BAL58 marine group
*Polaribacter* sp. MED152 (re-annotation)	2,606,217,529	2,961,474	2695	*Bacteroidetes*	*Polaribacter*
*Methylophilales* sp. HTCC2181	639,857,020	1,304,428	1377	*Betaproteobacteria*	OM43 clade
SAR86 cluster bacterium SAR86B	2,597,489,920	1,679,540	1890	*Gammaproteobacteria*	SAR86 clade
*Rhodobacterales* sp. HTCC2255 (original sequence, contaminants removed)	2,517,572,075	2,224,475	2209	*Alphaproteobacteria*	*Roseobacter* NAC11-7
*Formosa* sp. AK20	2,531,839,038	3,055,484	2841	*Bacteroidetes*	*Formosa*
Candidatus *Pelagibacter ubique* SAR11 HTCC1062 (re-annotation)	2,606,217,343	1,308,759	1393	*Alphaproteobacteria*	SAR11 clade
Marine gamma proteobacterium sp. HTCC2207 (re-annotation)	2,606,217,324	2,620,870	2388	*Gammaproteobacteria*	SAR92 clade
SAR86 cluster bacterium SAR86A	2,597,489,919	1,245,342	1340	*Gammaproteobacteria*	SAR86 clade
Candidatus *Pelagibacter ubique* SAR11 HTCC1002 (re-annotation)	2,606,217,624	1,327,604	1415	*Alphaproteobacteria*	SAR11 clade
*Formosa agariphila* KMM 3901	2,585,427,664	4,228,350	3630	*Bacteroidetes*	*Formosa*

Metatranscriptomic datasets were mapped on the 15 genomes and on the assembled contigs using Bowtie 2 version 2.2.4 (Langmead and Salzberg, 2012) with one mismatch in the seed and multiple hits reporting enabled for the metagenomic binning. Ribosomal RNA was removed from metatranscriptomic datasets prior to mapping employing SortMeRNA version 2.0 (Kopylova et al., 2012) (Table 5). The number of unique sequences per gene was calculated, and the overall mapping result was normalized by the total number of unique reads in the sample. The top 23,884 open reading frames based on number of assigned, normalized reads are provided in Supplementary Table S1.

**Table 5:** **Sequence statistics of the quality trimmed and rRNA depleted metatranscriptomic data sets used for mapping**.

**Sample**	**Number of sequences**	**Average read length**	**Total bps**	**Mapped reads**	**Mapping rate (%)**
1	4,398,462	73.66	3,23,999,530	3,905,801	88.80
3a	2,256,897	73.52	165,918,281	2,004,411	88.81
3b	3,058,087	73.69	225,347,730	2,538,870	83.02
4	1,536,922	72.64	111,646,018	1,238,340	80.57
5	1,675,015	87.74	146,961,679	1,475,942	88.12
6	1,281,015	72.90	93,392,214	1,102,985	86.10
9	8,352,509	84.56	706,301,999	7,416,227	88.79
10	4,976,491	90.96	452,673,412	4,236,320	85.13
13	15,976,940	72.28	1,154,891,997	13,685,288	85.66
15	2,028,509	72.70	147,476,064	1,662,304	81.95
Total	45,540,847	77.48	3,528,608,924	39,266,488	86.22

*Depletion was performed with SortMeRNA (Kopylova et al., 2012)*.

Results from the genome mapping were additionally normalized by the unique RNA/DNA ratio calculated for each bacterial group in the respective sample (see Campbell and Kirchman, 2013). In detail, the ratio was calculated by dividing the relative abundance of a 16S rRNA transcript by the relative abundance of the corresponding 16S rRNA gene. On the one hand, assuming that the major fraction of the bacterioplankton community is present outside and inside the algal bloom, the abundance at DNA level of single species is mainly linked to cell abundance rather than other factors such as 16S rRNA gene copy number. On the other hand, RNA abundance is correlated with protein synthesis (Blazewicz et al., 2013) and may indirectly serve as approximation for gene expression levels. Therefore, a high RNA/DNA ratio reflects an increased gene expression per cell and vice versa.

### Amplification and sequencing of 16S rRNA

To assess bacterial community structures, the V3–V6 region of the bacterial 16S rRNA was amplified by PCR. The PCR reaction (50 μl) contained 10 μl of five-fold Phusion HF buffer, 200 μM of each of the four desoxynucleoside triphosphates, 1.5 mM MgCl_2_, 4 μM of each primer, 2.5% DMSO, 2 U of Phusion high fidelity hot start DNA polymerase (Thermo Fisher Scientific), and approximately 50 ng of DNA or 25 ng of cDNA as template. The following thermal cycling scheme was used: initial denaturation at 98°C for 5 min, 25 cycles of denaturation at 98°C for 45 s, annealing at 60°C for 45 s, followed by extension at 72°C for 30 s. The final extension was carried out at 72°C for 5 min. Negative controls were performed by using the reaction mixture without template. The V3–V6 region was amplified with the following set of primers according to Muyzer et al. (1995) containing the Roche 454 pyrosequencing adaptors, keys and one unique MID per sample (underlined): 341f 5′-CCATCTCATCCCTGCGTG TCTCCGAC-TCAG-(dN)_10_-CCT ACGGRAGGCAGCAG-3′ and 1063r 5′-CCT ATCCCCTGTGTGCCTTGGCAGTC-TCAG-CTCACGRCACGAGCTGAC G-3′. Obtained PCR products were controlled for appropriate size and subsequently purified using the peqGOLD gel extraction kit (Peqlab) as recommended by the manufacturer. Three independent PCR reactions were performed per sample, purified by gel extraction, and pooled in equal amounts. Quantification of the PCR products was performed using the Quant-iT dsDNA HS assay kit and a Qubit fluorometer (Invitrogen™) as recommended by the manufacturer. The Göttingen Genomics Laboratory determined the sequences using a Roche GS-FLX++ 454 pyrosequencer with Titanium chemistry (Roche, Mannheim, Germany).

### Processing and analysis of 16S rRNA datasets

Generated 16S rRNA gene and rRNA datasets were processed as described by Wietz et al. (2015). In brief, sequences were preprocessed with QIIME and subsequently denoised employing Acacia (Bragg et al., 2012). Remaining primer sequences were truncated employing cutadapt (Martin, 2011). To remove chimeras, sequences were first dereplicated and putative chimeras were removed using UCHIME in *de novo* mode and subsequently in reference mode using the most recent SILVA database (SSURef 119 NR) as reference dataset (Edgar et al., 2011; Quast et al., 2013). Processed sequences of all samples were joined and clustered in operational taxonomic units (OTUs) at 3 and 20% genetic dissimilarity according to Wemheuer et al. (2013) employing the UCLUST algorithm with optimal flag (Edgar, 2010). To determine taxonomy, a consensus sequence for each OTU at 97% genetic similarity was classified by BLAST alignment against the Silva SSURef 119 NR database (Camacho et al., 2009). All non-bacterial OTUs were removed. Sequences statistics are shown in Table 6. The curated OTU table is provided as Supplemental Table S2. The final Alpha diversity indices were calculated with QIIME as described by Wemheuer et al. (2013) (see Table 7).

**Table 6:** **Statistics of the 16S rRNA analysis**.

**Sample**	Before preprocessing		After preprocessing		After denoising		After removal of non-bacterial or chimeric sequences	
	**No. of sequences**	**Average length**	**No. of sequences**	**Average length**	**No. of sequences**	**Average length**	**No. of sequences**	**Average length**
**DNA**								
1	10,692	705.0	10,486	676.1	10,172	673.9	6380	668.5
3a	12,328	704.6	12,155	674.4	11,814	673.7	6611	667.8
3b	13,234	705.6	13,086	674.9	12,771	674.1	7756	669.7
4	8589	710.5	8467	679.3	8283	678.9	4923	676.0
5	11,954	701.4	11,749	671.8	11,313	670.2	6435	661.9
6	8580	700.0	8466	671.3	8153	668.6	4557	658.1
9	8198	703.5	8088	673.6	7947	673.1	4265	666.1
10	5801	691.7	5726	664.0	5523	662.1	2946	645.3
13	9009	709.6	8904	678.9	8751	678.3	4463	672.4
15	3339	703.5	3306	673.4	3234	673.2	1789	668.4
Total	91,724	704.0	90,433	674.2	87,961	673.0	50,125	666.3
**RNA**								
1	7296	708.3	7178	678.1	6998	676.9	3099	671.5
3a	12,612	710.5	12,457	680.2	12,078	678.2	4806	670.6
3b	6601	695.8	6510	667.6	6240	664.8	2720	649.5
4	12,901	696.0	1268	668.9	1195	668.7	491	657.4
5	11,944	712.5	11,584	682.1	11,297	680.1	4588	673.2
6	14,831	703.7	14,642	674.7	14,443	674.3	5520	663.3
9	10,499	702.4	4945	673.1	4803	671.7	1836	657.9
10	9480	712.8	9336	682.3	9115	680.7	3489	671.8
13	4669	710.8	4515	680.3	4412	678.9	1795	670.5
15	9251	703.2	9044	674.1	8679	671.4	3638	659.8
Total	100,084	705.4	81,479	677.2	79,260	675.6	31,982	666.0

**Table 7:** **Alpha diversity indices at 97 and 80% genetic similarity derived from the 16S rRNA analysis**.

	Richness		Maximal number of OTUs		Coverage (%)		Chao1		Shannon (H^**′**^)	
	**97%**	**80%**	**97%**	**80%**	**97%**	**80%**	**97%**	**80%**	**97%**	**80%**
**DNA**										
1	143.3	18.5	223.6	20.2	64.1	91.4	333.5	22.9	4.15	2.25
3a	160.2	19.7	252.7	21.0	63.4	93.8	325.1	23.0	4.19	2.17
3b	147.2	18.3	229.9	19.9	64.0	91.8	292.2	22.5	3.80	2.04
4	135	20.3	186.6	21.3	72.4	95.3	326.1	26.2	4.40	2.37
5	185.4	23.1	318.6	25.3	58.2	91.5	443.6	25.3	4.47	2.35
6	177.4	23.5	288.3	24.9	61.5	94.3	423.3	30.8	4.72	2.42
9	150.7	22.4	221.2	23.9	68.1	93.7	378.5	32.4	4.49	2.38
10	146.2	20.2	223.4	21.6	65.4	93.5	360.6	22.6	4.13	2.03
13	156	24.2	252.4	27.3	61.8	88.6	374.8	34.9	4.35	2.14
15	136	18	216.1	19.0	62.9	94.6	379.1	18.0	3.91	2.14
**RNA**										
1	282.6	28.6	563.7	32.2	50.1	88.9	882.4	40.6	5.62	2.39
3a	252.9	23.1	482.0	24.9	52.5	92.7	816.4	28.8	5.21	2.26
3b	273	25.9	496.7	27.4	55.0	94.4	689.8	32.3	5.24	2.15
4	NA	NA	NA	NA	NA	NA	NA	NA	NA	NA
5	267.9	25	547.1	26.2	49.0	95.4	849.3	35.0	5.15	2.38
6	297.8	24.7	634.1	26.0	47.0	95.0	888.3	31.3	5.53	2.52
9	284.1	22	572.1	22.8	49.7	96.4	829.9	23.3	5.35	2.39
10	275.6	23.6	665.3	26.0	41.4	90.7	919.9	27.5	5.13	2.24
13	246.7	29	508.2	32.7	48.5	88.6	660.1	35.0	5.19	2.40
15	276	22	599.8	23.3	46.0	94.5	873.6	27.6	5.38	2.35

*OTU, operational taxonomic unit*.

### Statistical analysis

All statistical analyses were conducted employing R [version 3.1.2; R Core Team, 2014 (http://www.R-project.org/]. Possible correlations between phytoplankton bloom presence and richness (number of OTUs) as well Shannon indices, abundance, and gene expression were determined employing the non-parametric Wilcox rank-sum test (Gifford et al., 2013). Correlations were considered as significant with *P* ≤ 0.05. Sample 1 was excluded from the statistical analysis because it was taken in another bloom event.

### Sequence data deposition

Sequence data were deposited in the sequence read archive of the National Center for Biotechnology Information under accession numbers SRA061816 and SRA060677, respectively (for details see Table 3).

## Results and discussion

### Characteristics of the samples

In the current survey, we examined structural and functional responses of the bacterioplankton community toward a phytoplankton bloom. Samples for community analysis were taken randomly at different locations and different depths within a *P. globosa* bloom in the German Bight (Figure 1, Table 1). Six samples were taken in presence of the phytoplankton bloom (samples 5, 6, 9, 10, 13, and 15) and three in bloom absence (samples 3a, 3b, and 4). One sample was taken near the Weser river outfall (sample 1). Salinity ranged from 30.7 to 32.7 psu. Fluorescence was approximately 0.45 and 2.2 mg/m^3^ outside and inside the algal bloom, respectively. Temperatures ranged from 8.2 to 11.8°C. All environmental parameters are listed in Table 2. Based on our previous analysis, most measured parameters were significantly linked to algal bloom presence (see Wemheuer et al., 2014). Only the suspended particulate matter content (SPM) and the nitrite concentration exhibited no direct correlation to bloom presence.

### Bloom presence affects bacterial community structures

Total and active bacterioplankton community structures were assessed by pyrosequencing-based analysis of the V3–V6 region of the 16S rRNA amplified from environmental DNA and RNA, respectively. A total of 50,125 and 31,982 high-quality bacterial 16S rRNA sequences were obtained across all 10 samples at DNA and RNA level, respectively (Table 6). Calculated rarefaction curves (data not shown) as well as diversity indices revealed that the majority of the bacterial community was recovered by the surveying effort (Table 7).

**Figure 2:**
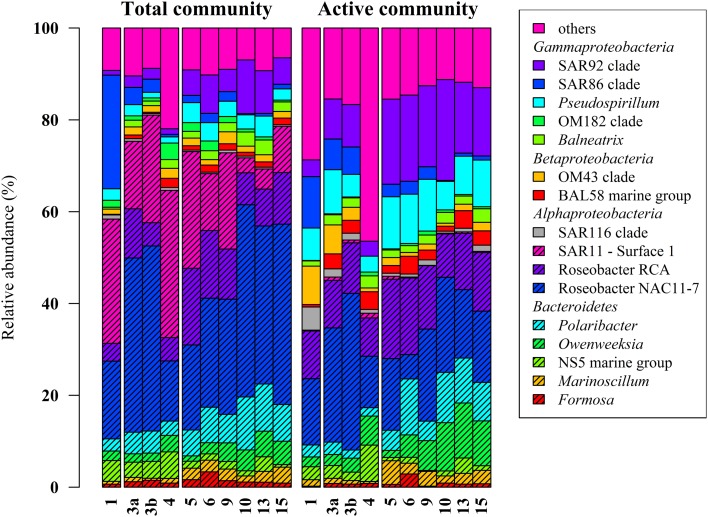
**Relative distribution of abundant bacterial lineages in the total (DNA-based) and active (RNA-based) bacterioplankton community at stations outside (1–4) and inside (5–15) the examined phytoplankton bloom**. Only groups with an average abundance of more than 1% either at DNA or RNA level are shown. Station 1 is separated from the oher samples because it was located in a bloom outside of the area of the examined bloom.

Classification of the obtained 16S rRNA sequences revealed that *Proteobacteria* and *Bacteroidetes* were the most abundant bacterial phyla across all samples (approximately 78% and 20%, respectively). At higher taxonomic resolution, the majority of the obtained sequences was affiliated to 17 bacterial groups, clades, and genera (Figure 2). These groups represented different lineages within the *Alpha*-, *Beta*-, and *Gammaproteobacteria* and the *Bacteroidetes*. These results are in accordance with our previous study (Wemheuer et al., 2014) and recent investigations of bacterial communities in the North Sea (Alderkamp et al., 2006; Sapp et al., 2007; Teeling et al., 2012). However, in our previous study, the number of *Bacteroidetes* was rather low which can be attributed to the differences in primer pairs and variable regions of the 16S rRNA gene used in our previous study.

Alphaproteobacteria accounted for 50% of all sequences with a higher abundance at DNA and RNA level (59 and 41%, respectively). The opposite was recorded for the Gammaproteobacteria, which accounted for 16% (DNA) and 31% (RNA), respectively. The increased abundance of the Gammaproteobacteria was mainly attributed to the higher abundances of Pseudospirrilii and the SAR92 clade. Changes in the abundances of the different alphapoteobacterial taxa were mainly attributed to the overall low abundance of the SAR11 clade at RNA level. A low activity of SAR11 is supported by other studies (West et al., 2008; Lamy et al., 2010; Klindworth et al., 2014). For example, Lamy et al. found an overall low abundance and activity of the SAR11 clade in a *P. globosa* bloom in the eastern English Channel. In another study, Alonso and Pernthaler (2006b) showed that SAR11 is highly abundant but not very active in costal North Sea waters. In addition, West et al. (2008) demonstrated that SAR11 was more abundant at DNA level than at RNA level in the Southern Ocean.

Several groups responded significantly toward algal bloom presence at DNA and/or RNA level, e.g., the abundance of the SAR92 clade was three times higher at RNA level and in bloom presence. This is in accordance with previous studies (Pinhassi et al., 2005; West et al., 2008; Klindworth et al., 2014; Wemheuer et al., 2014). For example, a phytoplankton bloom induced by inorganic nutrient enrichment influenced SAR92 in a mesocosm experiment (Pinhassi et al., 2005). Klindworth et al. (2014) found that members of the *Rhodobacteraceae* and SAR92 clade exhibited high metabolic activity levels during a bloom succession, which indicates their important role during bloom events. In addition, the 16S cDNA estimates for SAR11 were notably lower in the earlier bloom sample. The authors suggest that members of this clade could not profit from the increasing availability of nutrients in the decaying bloom and thus were outcompeted by other clades. This is in line with our study in which we found significant lower abundances of SAR11 in the bloom presence and at RNA level.

Members of the two genera Marinoscillum and Polaribacter were significantly more abundant in bloom samples both at DNA and RNA level. *Bacteroidetes* are widespread in marine systems and play an important role in organic matter degradation (Gómez-Pereira et al., 2010). The higher abundance of this phylum during the phytoplankton bloom was verified by recent findings (Alderkamp et al., 2006; Lamy et al., 2010; Tada et al., 2012; Teeling et al., 2012). The strongest increase in activity during the senescent stage of a *P. globosa* bloom in the North Sea was observed for *Bacteroidetes* (Alderkamp et al., 2006) A mesocosm experiment targeting bacterial succession patterns during a diatom bloom revealed that *Bacteroidetes* had a relatively high growth potential as the bloom peaked (Tada et al., 2012). The authors suggested that the early development contributed to the initial stage of bloom decomposition. Therefore, this phylum seems to benefit from the conditions provided by the algal bloom and might play an important role in the degradation of phytoplankton-derived organic matter. Klindworth et al. (2014) mapped metatranscriptomic data on assembled and taxonomically classified metagenomic data and found that Formosa and Polaribacter acted as major algal polymer degraders. A similar conclusion was drawn in a study of a *P. globosa* bloom in the eastern English Channel. Here, members of *Bacteroidetes* group dominated the activities and the abundances during the growth phase of the algae (Lamy et al., 2010).

### Bloom presence affects bacterioplankton gene expression

After removal of ribosomal RNA, nearly 45 million Illumina reads remained and were used for environmental gene expression analysis. Generated mRNA datasets were initially mapped on the 15 reference genomes belonging to abundant marine genera and lineages. However, only 10% of the sequences mapped to these reference genomes. Most of these sequences were affiliated to the genome of *P. temperata* RCA23 (see Supplementary Table S3). This strain was isolated in the German Wadden Sea (Giebel et al., 2011, 2013), and its genome was recently described (Voget et al., 2014).

Mapping mRNA datasets on genomes is a common approach when analyzing metatranscriptomic data (e.g., Gifford et al., 2013). The advantage of this approach is that community functions can be linked to a certain organism. However, most reads are not included in the analysis because reference genomes for many marine lineages are still missing. Another problem is the data normalization when mapping metatranscriptomic data on genomes. In a transcriptomic approach, all sequences derive from a single organism and data can be normalized by the number of reads mapped. However, in a metatranscriptomic approach, the amount of sequences affiliated to an organism is not only linked to its gene expression but also to the gene expression of all other community members. Thus, a decrease in abundance can be caused either by a lower gene expression of the organism or by an increased expression of other community members.

Here, we mapped the mRNA data on reference genomes representing marine lineages, which were abundant in the 16S rRNA gene analysis. These genomes included data from *P. temperata* RCA23 and HTCC2255, belonging to two *Roseobacter* lineages, the RCA and NAC11-7 clusters. Other reference genomes used were the genomes of HTCC2207 und MOLA455, both members of the SAR92 clade (Stingl et al., 2007; Courties et al., 2014). Mapping the datasets on assembled metagenomic and metatranscriptomic data resulted in an overall alignment rate of more than 86.22% (see Table 5). This overall high coverage is higher than in other studies. For example, Kopf et al. (2015) mapped up to 80% of their metatranscriptomic data on the corresponding metagenome. We were able to map our data on approximately 600,000 genes with almost 800,000 different functions.

**Figure 3:**
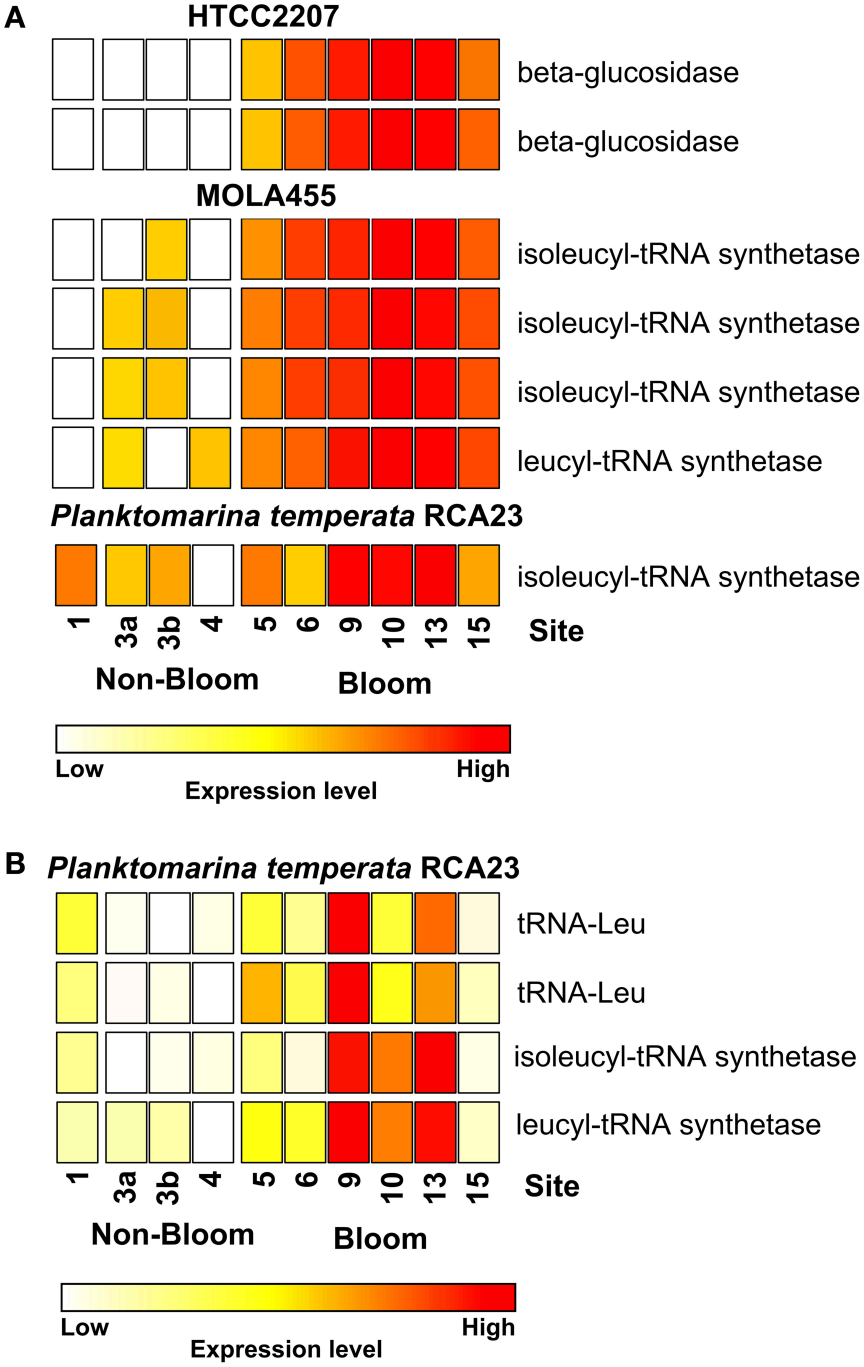
**Expression of certain gene involved in leucine/isoleucine incorporation and polymer degradation inside and outside the examined phytoplankton bloom**. Data either derives from the mapping on the assembled metagenomic and metatranscriptomic data (A) or from the mapping on reference genomes (B). Gene expressions levels are log2 transformed and standardized. To obtain only positive values, normalized gene expression values were increased by one prior to log transformation.

Most of the genes affiliated to the two members of the SAR92 clade were upregulated which corresponds to their increasing abundance at 16S rRNA transcipt level. For example, one leucyl-tRNA synthetase and three isoleucyl-tRNA synthetases affiliated to MOLA455 were significantly upregulated in the bloom (Figure 3A). Morover, two leucine-tRNAs were significantly upregulated in the bloom in *P. temperata* RCA23 (Figure 3B). In addition, an isoleucyl-tRNA synthetase affiliated to *P. temperata* RCA23 (Figure 3A) and a isoleucyl-tRNA synthetase and a leucyl-tRNA synthetase of *P. temperata* RCA23 (Figure 3B) were marginal significantly upregulated in the bloom (*P* < 0.1). This is in line with a study by West et al. (2008). The authors found that the *Roseobacter* groups NAC11-7 and RCA as well as the SAR92 clade were the most important contributors to leucine incorporation during the peak of a naturally iron-fertilized phytoplankton bloom in the Southern Ocean. This result is confirmed by a study about a *P. globosa* bloom in the English Channel (Lamy et al., 2010). Here, *Bacteroidetes* and *Gammaproteobacteria* were the most abundant and active groups during the growth period of the algae. *Gammaproteobacteria* and *Alphaproteobacteria* dominated by the *Roseobacter* clade accounted for the major part of leucine incorporation after the disappearance of the bloom. In addition, the contributions of different bacterial groups to bulk abundance and leucine incorporation were partly correlated with cell-specific exoproteolytic and exoglucosidic activities and with particulate organic carbon. This indicates some specificity of these bacterial groups with respect to their ecological role in the environment. Interestingly, we identified two beta-glucosidases affiliated to HTCC2207 being expressed only in bloom samples (Figure 3A). In the study of Teeling et al. (2012), metagenomic and metaproteomic data indicated the presence of distinct sets of carbohydrate-active enzymes (CAZymes) and transporters, which suggested a positive selection for bacteria with the capacity to decompose phytoplankton biomass. Four HTCC2207 indicator genes have been described to contain cadherin domains involved in complex carbohydrate degradation via cell aggregation and direct binding to cellulose, xylan, and related compounds (Gifford et al., 2013). This might explain the increase of the SAR92 clade as observed in the present study. In addition, the role of Gammprotaobacteria during polysaccharide degradation has been recently addressed in a study by Wietz et al. (2015). The authors showed that alginate and agarose degradation covaried with the abundance of different lineages within the *Gammaproteobacteria*.

Interestingly, a major fraction of the expressed genes affiliated to SAR92 clade and other bacterial lineages was linked to several genes such as RNA polymerases, heat shock proteins, chaperons, sigma factors, and ribosomal proteins. This is consistent with a study from Klindworth et al. (2014). In this study, the most abundant mRNA transcripts with known functions coded for housekeeping genes including DNA gyrase, elongation factors, and sigma factors. The authors indicated that this reflects differences in nutritional ecological strategies of the dominant bacterial classes.

However, the high number of heat shock and other stress-related genes overexpressed in bloom samples in members of the SAR92 clade might not necessarily reflect its ecological role as a polymer degrader but might also be caused by a higher stress tolerance toward the rapidly changing conditions during the phytoplankton bloom (Wemheuer et al., 2014). PON, Chl a, and phaeopigments of stations in the bloom area were significantly higher than that outside the bloom area. An increasing pH from 7.9 to 8.7 was observed as a result of CO_2_ net fixation into the alga during a phytoplankton bloom (Brussaard et al., 1996). Members of SAR92 clade might benefit from bloom conditions due to their high stress tolerance level rather than filling one of the specialized ecological niches formed during a phytoplankton bloom.

### Adaption to environmental changes by higher genome plasticity

**Figure 4:**
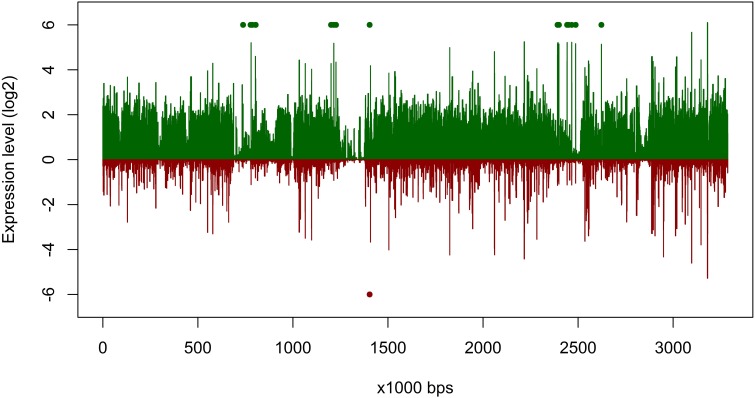
**Gene expression of *Planktomarina temperata* RCA 23 inside and outside the examined phytoplankton bloom**. Gene expressions are log2 transformed mean values from the three non-bloom and six bloom stations, respectively. To obtain only positive values, normalized gene expression values were increased by one prior to log transformation. Expression inside the bloom is depicted in green, outside the bloom in red. Green/red dots mark the position of transposases, which were highly upregulated.

The overall expression level of *P. temperata* RCA23 was higher inside the bloom (Figure 4). Numerous genes encoding for transposases in its genome were highly overexpressed. Transposases are the most abundant and most ubiquitous genes in nature (Aziz et al., 2010). In addition, investigation of the metatranscriptomic bins revealed the presence of several transposases that were affiliated to *P. temperata* and overexpressed in the bloom (Figure 4). It has been shown that some bacteria expressed transposases under changing environmental conditions to rearrange genome architecture. For example, up to 81 genes encoded for transposases were upregulated in *Microcystis aeruginosa* relative to the control when grown on urea (Steffen et al., 2014). Genome rearrangements and the resulting genome mosaics have been also found in other members of the *Roseobacter* clade. The genomes of *Octadecabacter arcticus* and *O. antarcticus* are highly different despite their similarity on 16S rRNA gene sequence level and the presence of some unique gene features (Vollmers et al., 2013). This is attributed to genomic rearrangements caused by an unusually high number of transposases in the genomes of both *Octadecabacter* strains. We assume that the recorded overexpression could result in a higher genome plasticity/heterogeneity of this population and thus might be a possible adaptation strategy of *P. temperata* to environmental changes. Moreover, as found in other members of the *Roseobacter* clade, it might be one of the key features of this group explaining its high abundance in marine ecosystems and its ability to adapt to various marine niches. However, comparative genome studies are missing because only one genome of the genus *Planktomarina* is currently available. Consequently, this issue cannot been fully answered yet.

## Conclusions

Active bacterial communities in the North Sea are dominated by only a few marine groups such as the *Roseobacter* RCA cluster. Some of these lineages responded significantly toward the *P. globosa* bloom investigated in this study. For example, the SAR92 clade was three times more abundant at active bacterial community level and in bloom presence. The metatranscriptomic approach revealed that these groups are not dominated by well-studied isolates or type species as only 10% of all metatranscriptomic sequences mapped on reference genomes. Therefore, *in situ* experiments employing available isolates do not necessarily reflect environmental conditions and, thus, only provide limited information on the ecological role of the studied isolates. However, mapping these reads on assembled metagenomic and metatranscriptomic sequences led to an overall mapping rate of more than 85% demonstrating the power of this combined approach. The functional analysis performed in this study provides insights into gene expression patterns of the abundant community members. The high abundance of the SAR92 clade, which is supposed to be involved in polymer-degradation during and after the bloom, is attributed to a higher stress tolerance indicated by the high number of heat shock expressed in the bloom. Although the number of field studies targeting the active bacterial community either by metatranscriptomic or metaproteomic approaches has been increased over the past years, the complex dynamics of marine environments are still largely unexplored. This study provides a deep insight into structural and functional responses of the bacterioplankton community toward a phytoplankton bloom. Therefore, it paved the way for a better understanding of the complex dynamics of marine bacteria and their interactions with the surrounding environment.

## Author contributions

RD and BW conceived and designed the experiments; BW, FW, JH, SV, and FM performed the experiments and analyzed the data; BW, FW, and RD wrote the paper; all authors reviewed, edited, and approved the manuscript.

### Conflict of interest statement

The authors declare that the research was conducted in the absence of any commercial or financial relationships that could be construed as a potential conflict of interest.
